# Supplementary material for: The interactome of intact mitochondria by cross-linking mass spectrometry provides evidence for coexisting respiratory supercomplexes
Source: Mol Cell Proteomics. 2017 Dec 8;17(2):216–32. doi: 10.1074/mcp.RA117.000470 (PMC5795388; doi:10.1074/mcp.RA117.000470)
Supplement: Supplemental Data [file supp_RA117.000470_133922_0_supp_23978_fzffwf.zip › spectra_annotation/mito_DR_spectra_annotation/257-1-15-1-5-1.pdf]

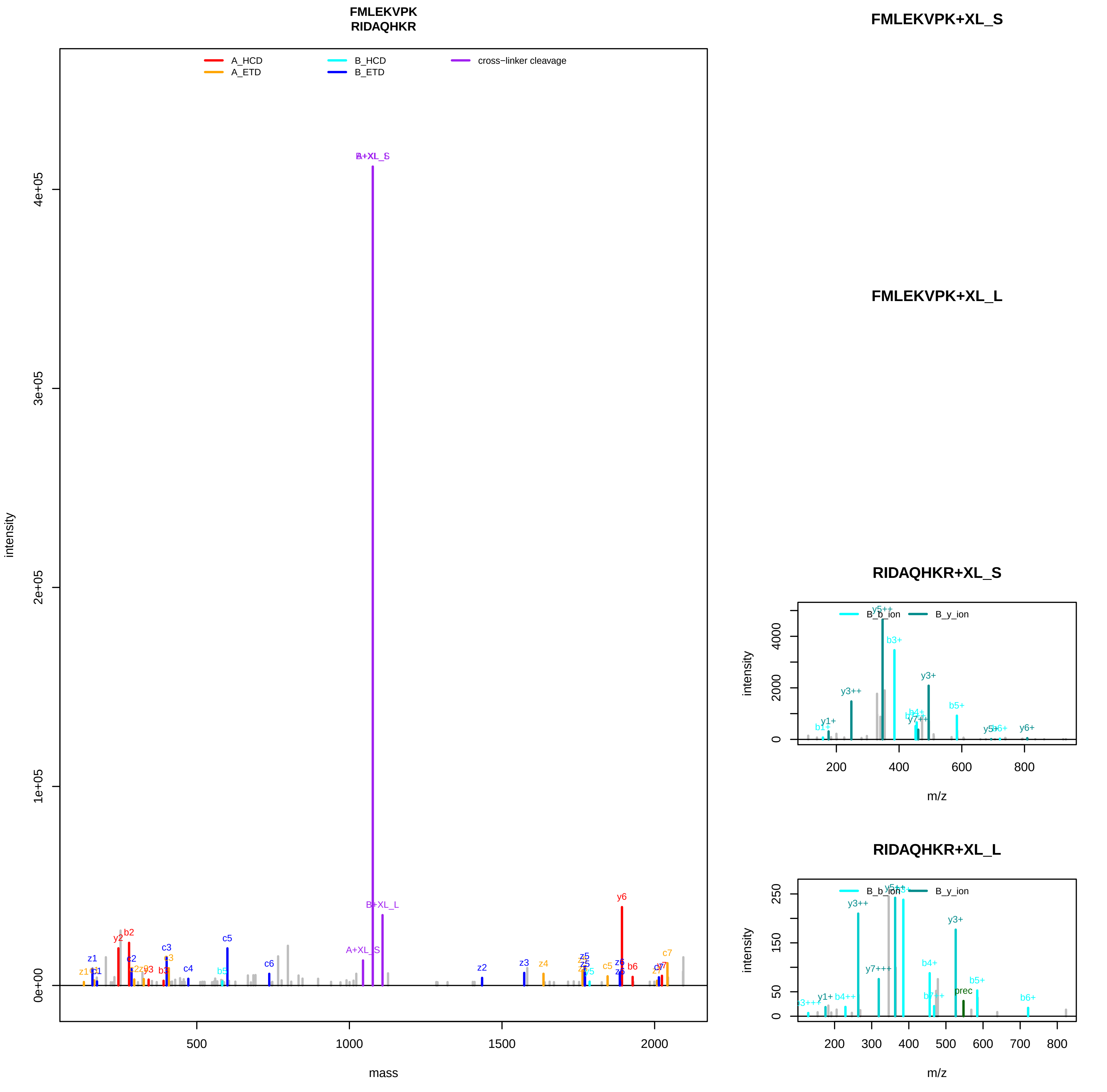
FMLEKVPK
RIDAQHKR
A_HCD
A_ETD
B_HCD
B_ETD
cross−linker cleavage
A+XL_S
B+XL_L
A+XL_S
y6
y2
b2
c2
c3
c5
c4
c6
z1
c1
b3
b5
z2
z3
z4
z5
c5
z6
b6
c7
intensity
mass
0e+00
1e+05
2e+05
3e+05
4e+05
500
1000
1500
2000
FMLEKVPK+XL_S
FMLEKVPK+XL_L
RIDAQHKR+XL_S
B_b_ion
B_y_ion
y5++
b3+
y3+
y3++
b5+
y1+
b1+
b4+
y6+
intensity
m/z
0
2000
4000
200
400
600
800
RIDAQHKR+XL_L
B_b_ion
B_y_ion
y5++
y3++
y3+
y7+++
b4+
b5+
y1+
b4++
prec
b6+
intensity
m/z
0
50
150
250
200
300
400
500
600
700
800
